# Supplementary material for: Sex-Sparing Robot-Assisted Radical Cystectomy with Intracorporeal Padua Ileal Neobladder in Female: Surgical Technique, Perioperative, Oncologic and Functional Outcomes
Source: J Clin Med. 2020 Feb 20;9(2):577. doi: 10.3390/jcm9020577 (PMC7073846; doi:10.3390/jcm9020577)
Supplement: Supplementary file 1 [file jcm-09-00577-s001.zip › Supplementary Table S1.docx]

**Supplementary Table 1. Health-related Quality of life assessment (EORTC QLQ-C30 questionnaire).**

|  | **Baseline** | **3-mo** | **1-year** | ***p* Value** |
| --- | --- | --- | --- | --- |
| Global health status/QoL | 42 (42–75) | 75 (67–83) | 83 (67–83) | **0.007** |
| Physical functioning | 100 (100–100) | 93 (87–93) | 93 (87–100) | **<0.001** |
| Role functioning | 100 (83–100) | 100 (67–100) | 100 (100–100) | 0.10 |
| Emotional functioning | 50 (42–92) | 83 (67–92) | 92 (83–100) | **0.04** |
| Cognitive functioning | 100 (100–100) | 100 (100–100) | 100 (100–100) | 0.78 |
| Social functioning | 100 (67–100) | 100 (67–100) | 100 (83–100) | 0.37 |
| Fatigue | 11 (0–22) | 22 (10–43) | 11 (0–33) | 0.61 |
| Nausea and vomiting | 0 (0–17) | 0 (0–0) | 0 (0–0) | 0.18 |
| Pain | 0 (0–0) | 0 (0–17) | 0 (0–17) | 0.26 |
| Dyspnea | 0 (0–0) | 0 (0–0) | 0 (0–0) | - |
| Insomnia | 0 (0–33) | 0 (0–33) | 0 (0–33) | 0.15 |
| Loss of appetite | 0 (0–0) | 0 (0–0) | 0 (0–0) | 0.60 |
| Constipation | 0 (0–0) | 0 (0–33) | 0 (0–33) | 0.61 |
| Diarrhoea | 0 (0–0) | 0 (0–0) | 0 (0–0) | 0.36 |
| Financial difficulties | 0 (0–0) | 0 (0–33) | 0 (0–33) | 0.15 |

For the functional items, the higher score represents a higher level of functioning. For the symptom items, a higher score means a higher level of symptomatology/problems. Data reported as median values (IQR). Friedman test was used.
