# Supplementary material for: Sex-Sparing Robot-Assisted Radical Cystectomy with Intracorporeal Padua Ileal Neobladder in Female: Surgical Technique, Perioperative, Oncologic and Functional Outcomes
Source: J Clin Med. 2020 Feb 20;9(2):577. doi: 10.3390/jcm9020577 (PMC7073846; doi:10.3390/jcm9020577)
Supplement: Supplementary file 1 [file jcm-09-00577-s001.zip › Supplementary Table S2.docx]

**Supplementary Table S2.** Bladder-cancer-specific quality of life EORTC QLQ- BLM30 questionnaire.

|  | **Baseline** | **3-mo** | **1-year** | ***p* Value** | ****p* Value** |
| --- | --- | --- | --- | --- | --- |
| Urinary symptoms and problems | 0 (0–9) | 14 (0–24) | 5 (0–20) | **0.02** | **0.08** |
| Future perspective | 67 (33–67) | 33 (22–67) | 22 (11–44) | 0.10 |  |
| Abdominal bloating/flatulence | 0 (0–0) | 0 (0–17) | 0 (0–17) | 0.10 |  |
| Body image | 10 (0–11) | 0 (0–22) | 0 (0–22) | 0.72 |  |

A higher score indicates a higher level of symptomatology/problems. Data reported as median values (IQR). Friedman test was used. **p* value-Comparison between baseline and 1-year data.
