# Supplementary material for: Sex-Sparing Robot-Assisted Radical Cystectomy with Intracorporeal Padua Ileal Neobladder in Female: Surgical Technique, Perioperative, Oncologic and Functional Outcomes
Source: J Clin Med. 2020 Feb 20;9(2):577. doi: 10.3390/jcm9020577 (PMC7073846; doi:10.3390/jcm9020577)
Supplement: Supplementary file 1 [file jcm-09-00577-s001.zip › Supplementary Table S3.docx]

**Supplementary Table S3.** Female Sexual Function Index (FSFI) questionnaire.

|  | **Baseline** | **3-mo** | **1-Year** | ***p* Value** | ****p* Value** |
| --- | --- | --- | --- | --- | --- |
| ***FSFI*** | 31.9 (26.3–33) | 20.3 (6.8–30.9) | 26.2 (6.8–33) | **0.001** | **0.02** |
| ***Desire*** | 4.8 (3.6–5.4) | 3.6 (1.8–5.4) | 4.2 (1.8–5.4) | 0.057 |  |
| ***Arousal*** | 5.1 (3.9–5.7) | 3.3 (0–5.1) | 4.2 (0–5.1) | **0.02** | 0.10 |
| ***Lubrication*** | 5.4 (3.6–5.7) | 3.3 (0–5.4) | 5.1 (0–5.4) | **0.005** | **0.014** |
| ***Orgasm*** | 4.7 (4–5.2) | 3.2 (0–4.8) | 4.4 (0–5.2) | **0.04** | 0.10 |
| ***Satisfaction*** | 5.2 (4.8–6) | 3.6 (1.2–5.2) | 4 (0.8–6) | **0.006** | **0.025** |
| ***Pain*** | 6 (6–6) | 4.8 (0–5.6) | 5.2 (0–6) | **0.002** | **0.005** |

Data reported as median values (IQR). Friedman test was used. **p* value-Comparison between baseline and 1-year data.
