# Supplementary material for: Sex-Sparing Robot-Assisted Radical Cystectomy with Intracorporeal Padua Ileal Neobladder in Female: Surgical Technique, Perioperative, Oncologic and Functional Outcomes
Source: J Clin Med. 2020 Feb 20;9(2):577. doi: 10.3390/jcm9020577 (PMC7073846; doi:10.3390/jcm9020577)
Supplement: Supplementary file 1 [file jcm-09-00577-s001.zip › Supplementary Table S4.docx]

**Supplementary Table S4.** Baseline and clinical characteristics of Sex-sparing and Standard RARC.

| ***Patients, n*** | ***Sex-Sparing***  ***RARC (11)*** | ***Standard***  ***RARC (36)*** | ***p Value*** |
| --- | --- | --- | --- |
| **Age, year, mean (±SD)** | 47.1 (13) | 61.7 (9.2) | **<0.001** |
| **BMI, mean (±SD)** | 23.1 (3.3) | 24.4 (3.3) | 0.27 |
| **ASA score, *n* (%)**  **1**  **2**  **3**  **4** | 4 (36.4)  6 (54.6)  1 (9)  - | 4 (11.1)  26 (77.8)  4 (11.1)  - | 0.14 |
| **Preoperative eGFR, ml/min, mean (±SD)** | 84 (26.8) | 82.5 (24.5) | 0.87 |
| **Preoperative Hgb, g/dl, mean (±SD)** | 12.6 (1.9) | 12.6 (1.7) | 0.96 |
| **Neoadjuvant CHT, *n* (%)** | 4 (36.3) | 14 (38.8) | 0.82 |
