# Supplementary material for: Sex-Sparing Robot-Assisted Radical Cystectomy with Intracorporeal Padua Ileal Neobladder in Female: Surgical Technique, Perioperative, Oncologic and Functional Outcomes
Source: J Clin Med. 2020 Feb 20;9(2):577. doi: 10.3390/jcm9020577 (PMC7073846; doi:10.3390/jcm9020577)
Supplement: Supplementary file 1 [file jcm-09-00577-s001.zip › Supplementary Table S5.docx]

**Supplementary Table S5.** Perioperative, pathologic and functional characteristics of Sex-sparing and Standard RARC.

| ***Patients, n*** | ***Sex-Sparing***  ***RARC (11)*** | ***Standard***  ***RARC (36)*** | ***p Value*** |
| --- | --- | --- | --- |
| **Operative time, min, mean (±SD)** | 314.9 (93.1) | 315 (63.2) | 0.99 |
| **Hgb at discharge, g/dL, mean (± SD)** | 9.9 (2.2) | 10.4 (1.2) | 0.35 |
| **Hospital stay, days, mean (±SD)** | 10.5 (5.7) | 9.8 (4.3) | 0.67 |
| **Complications**  ***Clavien Low grade (1–2)***  ***Clavien High grade (≥3)*** | 4 (36.3)  4 (36.3)  0 (0) | 7 (19.4)  6 (16.7)  1 (2.7) | 0.25  0.21  0.57 |
| **pT stage, n (%)**  **0, a, is**  **1**  **2**  **3**  **4** | 6 (54.6)  1 (9.1)  -  4 (36.3)  - | 19 (52.8)  4 (11.1)  4 (11.1)  9 (25)  - | 0.35 |
| **pN stage, n (%)**  **0**  **1**  **2** | 11(100)  -  - | 31 (86.2)  1 (2.7  4 (11.1) | 0.43 |
| **Lymphnode count, mean (±SD)** | 26.2 (14.3) | 29.6 (11.6) | 0.51 |
| **Positive surgical margins, n (%)** | 0 (0) | 0 (0) | 1.00 |
| **Last eGFR, ml/min, mean (± SD)** | 79.2 (23.7) | 65.6 (20.8) | 0.43 |
| **Ureteroileal strictures, pts (%)** | 1 (9) | 4 (11.1) | 0.67 |
| **Neobladder stones, *n* (%)** | 1 (9) | 3 (8.3) | 0.93 |
| **Need for Intermittent self-catheterization, n (%)** | 3 (27.2) | 4 (11.1) | 0.12 |
