# Supplementary figures and images for: Sex-Sparing Robot-Assisted Radical Cystectomy with Intracorporeal Padua Ileal Neobladder in Female: Surgical Technique, Perioperative, Oncologic and Functional Outcomes
Source: J Clin Med. 2020 Feb 20;9(2):577. doi: 10.3390/jcm9020577 (PMC7073846; doi:10.3390/jcm9020577)

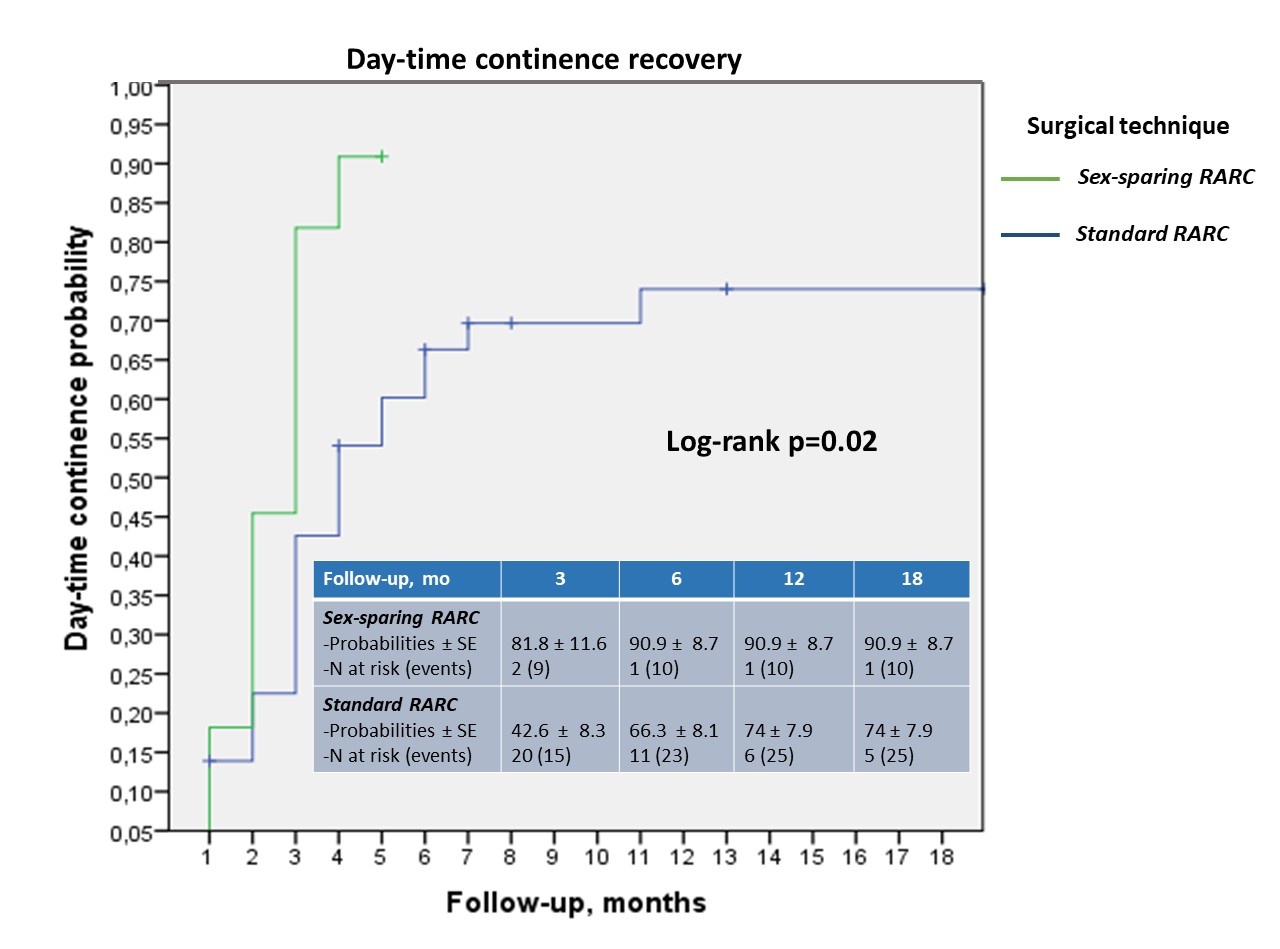

Supplement: Supplementary file 1 [file jcm-09-00577-s001.zip › Supplementary Figure S1.jpg]
